# Evaluation of Joint Formation and Mechanical Performance of the AA7075-T6/CFRP Spot Joints Produced by Frictional Heat

**DOI:** 10.3390/ma12060891

**Published:** 2019-03-17

**Authors:** Natalia Manente André, Jorge F. dos Santos, Sergio T. Amancio-Filho

**Affiliations:** 1 Department of Solid State Joining Processes, Institute of Materials Research, Materials Mechanics, Centre for Materials and Coastal Research, Helmholtz-Zentrum Geesthacht, 21502 Geesthacht, Germany; natalia.manente@hzg.de (N.M.A.); jorge.dos.santos@hzg.de (J.F.d.S.); 2 Institute of Materials Science, Joining and Forming, Graz University of Technology–TU Graz, BMVIT Endowed Professorship for Aviation, Kopernikusgasse 24/1, 8010 Graz, Austria

**Keywords:** Friction Spot Joining, aluminium alloys, fibre reinforced composites, friction, mechanical properties

## Abstract

The development of lightweight hybrid metal–polymer structures has recently attracted interest from the transportation industry. Nevertheless, the possibility of joining metals and polymers or composites is still a great challenge. Friction Spot Joining (FSpJ) is a prize-winning friction-based joining technique for metal–polymer hybrid structures. The technology is environment-friendly and comprises very short joining cycles (2 to 8 s). In the current work, aluminum alloy 7075-T6 and carbon-fiber-reinforced polyphenylene sulfide (CF-PPS) friction spot joints were produced and evaluated for the first time in the literature. The spot joints were investigated in terms of microstructure, mechanical performance under quasi-static loading and failure mechanisms. Macro- and micro-mechanical interlocking were identified as the main bonding mechanism, along with adhesion forces as a result of the reconsolidated polymer layer. Moreover, the influence of the joining force on the mechanical performance of the joints was addressed. Ultimate lap shear forces up to 4068 ± 184 N were achieved in this study. A mixture of adhesive–cohesive failure mode was identified, while cohesive failure was dominant. Finally, a qualitative comparison with other state-of-the-art joining technologies for hybrid structures demonstrated that the friction spot joints eventually exhibit superior/similar strength than/to concurrent technologies and shorter joining times.

## 1. Introduction

Interest has grown in the transport industry to use fiber-reinforced polymers aiming at reducing weight and fuel consumption in vehicles [1]. Glass- and carbon-fiber-reinforced polymers present optimal specific strength and stiffness, along with improved corrosion properties when compared with conventional materials such as steel [2]. Most of the time, the manufacturing of monolithic structures is not feasible due to technical and economic concerns [2]. Therefore, there is a growing trend of combining lightweight metal alloys with advanced fiber-reinforced polymers in the development of metal–polymer hybrid structures.

Over the past 30 years, aircraft manufacturers have been increasing the use of polymer composites in their products. Some well-known examples include the Boeing 787 (50% in weight composed of composites) [3], the Airbus A350 (53% in weight composed of composites) [4], and recently, the Embraer KC-390 that used polymer composites as a ballistic solution in a military model [5].

Mechanical fastening and adhesive bonding are the traditionally applied techniques to join metal–polymer hybrid structures in production lines [6]. However, there have been disadvantages related to stress concentration and additional process steps (for mechanical fastening) and long curing times (for adhesive bonding) which urged the development of alternative joining technologies [7]. Ultrasonic [8,9], resistance [10], induction [11], and laser [12] welding have been studied in the past years as advanced joining methods for metal–polymer hybrid structures. The present work considers frictional heat as the heat source for joining such dissimilar materials and presents Friction Spot Joining as a joining solution for metal–polymer hybrid structures.

Friction Spot Joining (FSpJ) is a friction-based method for joining metals to polymers or composites [13]. FSpJ produces high-quality joints relying on short joining cycles (2 to 8 s) and absence of filler material and post-joining treatment. Low-cost machinery and easy reparability are other advantages of this process. The feasibility of FSpJ has been demonstrated for several combinations of materials such as AZ31-O/glass-fiber- and carbon-fiber-reinforced polyphenylene sulfide (GF- and CF-PPS) [14] and AA6181-T4/CF-PPS [15] for automotive applications, and AA2024-T3/CF-PPS [16] for aerospace applications. Recently, the process was also demonstrated for carbon nanotube polycarbonate nanocomposites/AA6082-T6 single-lap joints for indirect heating of polymeric parts and electrostatic painting of metal–polymer hybrid parts in the automotive industry [17].

In the current study, AA7075-T6/CF-PPS friction spot joints were produced and evaluated for the first time in the literature. This combination of materials is strategic for aerospace applications due to the improved stress–corrosion cracking resistance of AA7075-T6 when compared with other aluminum alloys like AA2024-T3 [18]. The fundamentals of joint formation and the influence of the joining force on the mechanical performance of AA7075-T6/CF-PPS friction spot joints were addressed. The joint interface and bonding mechanisms were analyzed by optical and confocal laser scanning microscopy. The mechanical performance of the joints produced with three different joining forces was evaluated under quasi-static loading by using a lap shear test. A qualitative comparison of the quasi-static mechanical performance for metal–polymer or composite structures produced with different methods was also presented. Finally, the failure micro-mechanisms of the joints were briefly discussed.

## 2. Friction Spot Joining (FSpJ)

Friction Spot Joining uses a non-consumable tool composed of three pieces: a pin and a sleeve which rotate and move axially, and a stationary clamping ring [14]. The three pieces are mounted coaxially and have independent movements. The parts to be joined are aligned in an overlap configuration and then clamped between the backing plate and the clamping ring to ensure intimate contact during the process.

**Figure 1:**
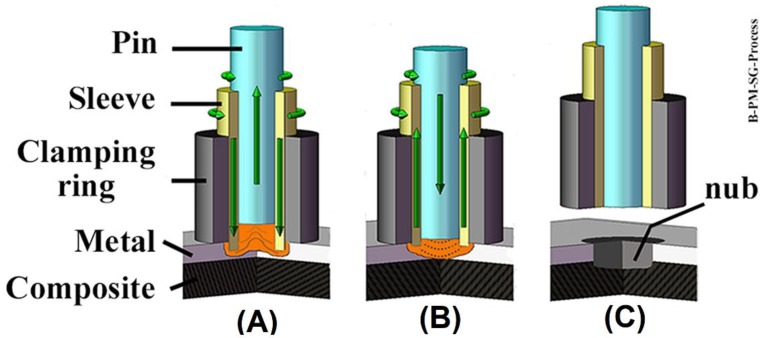
Description of the FSpJ process in three steps: (**A**) sleeve plunging, softening and deformation of the metal part; (**B**) spot refilling; (**C**) retraction of the tool and joint consolidation. (Adapted from Reference [14]).

The joining process can be divided into three steps. In the first step, the sleeve starts to rotate and plunges into the upper sheet (a metal sheet in this work, Figure 1A). Note, that to avoid the thermal–mechanical degradation of the polymer matrix and damage to the fiber reinforcement of the composite, the plunge of the sleeve is restricted to the metal part. The motion of the rotating sleeve in contact with the metal part generates frictional heat. Consequently, a volume of metal near the tool is softened and plastically deformed due to a local increase in temperature [15,16]. Concurrently with the sleeve plunging event, the pin is retracted forming a reservoir, which is filled with the softened metal (Figure 1A). In the second step of the process, the sleeve and pin move back to the metal surface. Thus, the softened metal is forced back into the metal part by the pin movement, thereby closing the keyhole left by the sleeve plunging (Figure 1B). In the third and final step, the tool is retracted from the surface of the metal part and the joint is kept clamped to consolidate under pressure (Figure 1C). The main process parameters of FSpJ are: the rotational speed of the tool (RS), the plunge depth of the sleeve into the metal part (PD), plunging and retracting time of the sleeve combined as the joining time (JT), and the joining force applied to the clamping ring during the process (JF) [19].

## 3. Materials and Methods

### 3.1. Aluminum Alloy 7075-T6

Aluminum alloy 7075 in the T6 temper condition (2-mm thick rolled sheets) was used in the current study. As the main alloying element, zinc provides high strength to this aluminum alloy through precipitation hardening. The addition of chromium improves the stress–corrosion cracking resistance of this alloy when compared with the 2XXX alloys [20]. The nominal chemical composition of the AA7075-T6 is presented in Table 1. A selection of relevant physical and mechanical properties of the alloy used in this work are listed in Table 2.

### 3.2. Carbon-Fiber-Reinforced Polyphenylene Sulfide (CF-PPS)

Carbon-fiber-reinforced polyphenylene sulfide (CF-PPS), a quasi-isotropic laminate, was used as the composite part in this work. Moreover, 2.17-mm-thick sheets with 43 wt.% carbon fiber-woven fabric (5H satin configuration) were selected. The carbon fiber fabric reinforcement is stacked as seven plies in the [(0.90)/(±45)]3/(0.90) sequence. CF-PPS is a high-performance thermoplastic composite that presents a continuous service temperature around 100 °C [22]. It was produced by TenCate (Netherlands). Several aerospace applications, such as the “J-Nose” subframe wings of Airbus A380 and the engine pylon cover of Airbus A340-500/600 are addressed for this material [22]. Here a selection of relevant physical and mechanical properties of CF-PPS is listed in Table 3.

### 3.3. Experimental Procedure

#### 3.3.1. Joining Procedure

Before the joining process, the aluminum part was sandblasted to increase its surface roughness. As reported in previous investigations [16,23], such mechanical surface pre-treatment improves the adhesion between aluminum and composite. Corundum (Al_2_O_3_) was used as a blasting medium with an average particle size of 100–150 µm. The samples were sandblasted for 10 s at a distance of 20 cm and an incidence angle of 45° of the blasting pistol. An average roughness (Ra) of 6.7 ± 0.4 µm was achieved.

**Figure 2:**
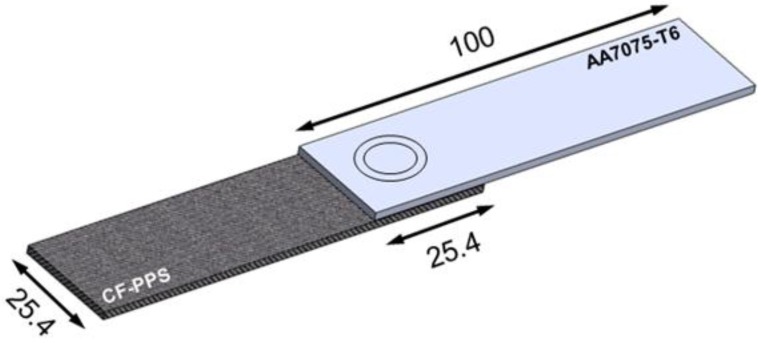
Configuration and dimensions of the joints (in mm).

Single overlap joints were produced using an FSp-joining equipment (RPS 200 Harms&Wende, Hamburg, Germany). The configuration and dimensions of the joints are shown in Figure 2.

The joining parameters used to produce the joints in this study are presented in Table 4. These joining parameters were obtained from the statistical analysis (full factorial design of experiments combined with analysis of variance) applied to maximize the ultimate lap shear force of the joints. Although the details of the statistical analysis for process optimization will be published in a separate document, the process parameter range used in the design of the experiments for the study to determine the range of optimal joining parameters of the current manuscript was: rotational speed—1900 to 2900 rpm; plunge depth—0.8 to 1.0 mm; joining time—4 to 8 s; and joining force—4 to 8 kN. To address the influence of the JF on the mechanical performance of the joints, RS, PD and JT were kept constant and a range of JF was investigated in this work.

**Figure 3:**
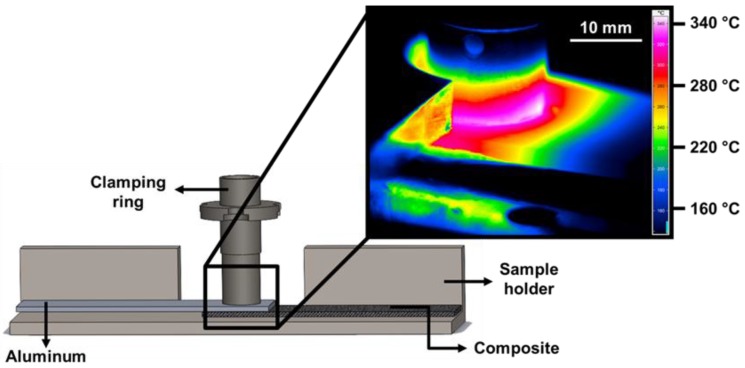
Schematic illustration of the set-up for infrared thermography and an example of the snapshot during the measurement.

The temperature evolution on the aluminum surface was monitored during the joining process using an infrared thermo-camera (High-end Camera Series ImageIR, Infratech GmbH, Dresden, Germany). The measurement was set within the range of 150–700 °C with a frequency of 20 Hz. The specimens were covered with a black and high-temperature-resistant paint prior to the joining process to avoid deviations regarding the emissivity of the aluminum alloy. Figure 3 shows a schematic example of the set-up for infrared thermography. The peak process temperature was considered as the maximum temperature identified on the aluminum surface.

#### 3.3.2. Microstructural Analysis

The joints were cut close to the middle of the spot and prepared for microstructural analysis, following standard grinding and polishing procedures. Optical (DM IR microscope, Leica, Wetzlar, Germany) and confocal laser scanning (VK-9700, Keyence, Osaka, Japan) microscopy were employed to investigate the microstructure and interface of the joints.

#### 3.3.3. Mechanical Testing

Lap shear testing under tensile loading was used to assess the quasi-static mechanical performance of the joints. The mechanical testing was performed according to the ASTM D3163-01 standard procedure by using a universal testing machine Zwick/Roell 1478 (Zwick Roell, Ulm, Germany). The cross-head speed of 1.27 mm min^−1^ was selected and the tests were performed at room temperature. Specimens with dimensions of 100 × 25.4 mm and 645.2 mm^2^ of overlap area were tested (Figure 2). The average ultimate lap shear force (ULSF) of the joints was evaluated based on three replicates for each joining condition. The strength of these joints was calculated by using the area of the external sleeve diameter (9 mm) as the nominal bonded area of the joints.

#### 3.3.4. Fracture Surface Analysis

The fractured surfaces of the joints were gold-sputtered and analyzed by scanning electron microscopy (SEM) (FEI, QUANTA FEG 650, Hillsboro, OR, USA). A voltage of 5 kV and a working distance of 17 mm were utilized. Confocal laser scanning microscopy (Keyence, Japan) was also used to generate 3D images of the fracture surface of the joints to estimate the volume of the composite entrapped into the aluminum part after the joining.

## 4. Results and Discussion

### 4.1. Temperature Evolution

**Figure 4:**
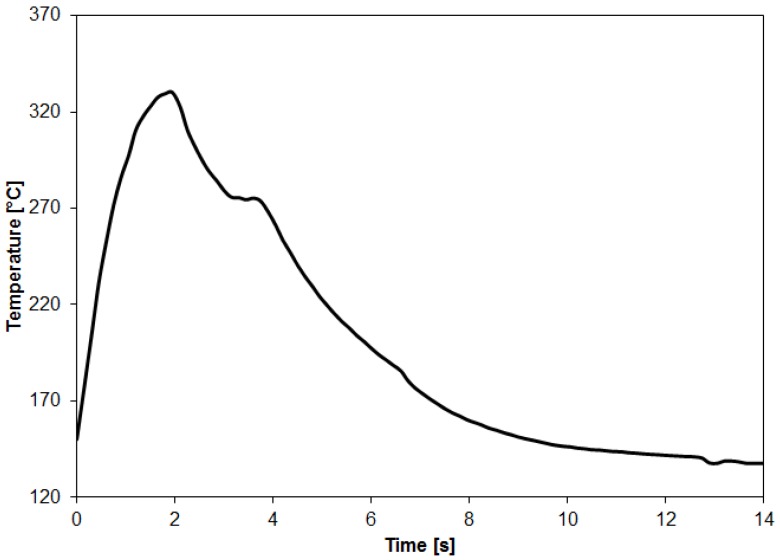
A representative curve of the temperature evolution on the aluminum surface during the FSpJ process (RS: 1900 rpm, PD: 0.8 mm, JT: 4 s, JF: 6 kN).

Figure 4 presents a representative curve of the temperature evolution on the aluminum surface during the FSpJ process. Considering the parameters used in this study, the maximum aluminum surface temperature achieved during the joining process was 331 ± 4 °C. On the one hand, such temperature represents about 77% of the incipient melting point of the AA7075-T6 [20]. Therefore, the metallic part of the joint is not expected to melt. Nevertheless, metallographic phenomena, such as recovery and dynamic recrystallization, are likely to occur due to the combination of the high temperature (0.77T_m_) and the shear rate applied by the rotating sleeve, as commonly observed in the friction-based welding processes [14,24]. On the other hand, the maximum temperature achieved is well above the T_g_ (120 °C) and T_m_ (280 °C) of the PPS matrix of the composite. Thus, it is expected that a thin layer of the PPS matrix close to the joint’s interface is melted during the joining process. The onset temperatures for the cross-linking (500 °C [25]) and chain scission (550° [25]) of PPS were not reached during the FSpJ process in this study. Therefore, extensive thermo-mechanical degradation of the polymeric part is not expected.

### 4.2. Joint Formation

**Figure 5:**
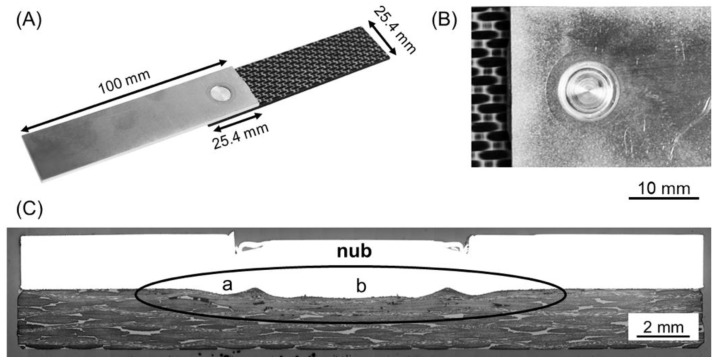
(**A**) Example of an AA7075-T6/CF-PPS friction spot joint along with typical (**B**) top view and (**C**) cross-section of the joints. The metallic nub is indicated with an ellipse in (**C**). The details of regions a and b are presented in Figure 6. (RS: 1900 rpm, PD: 0.8 mm, JT: 4 s, JF: 6 kN).

Figure 5A,B show a typical AA7075-T6/CF-PPS single lap joint along with its top view. Excellent surface finishing was achieved. The area where the sleeve plunge occurred has a bright and flat surface, as shown in Figure 5B.

A representative example of the cross-section of the joints is shown in Figure 5C. One notes that at the center of the joint a certain volume of the aluminum, which softened during the joining process, has symmetrically plastically deformed into the composite part because of the axial movement of the tool. This metallic undercut, known as the “metallic nub”, is responsible for the macro-mechanical interlocking between aluminum and composite. The metallic nub is a characteristic of FSp joints which was also observed in other combination of materials; it leads to macro-mechanical interlocking as one of the main bonding mechanisms in friction spot joints [14,15,16,26].

**Figure 6:**
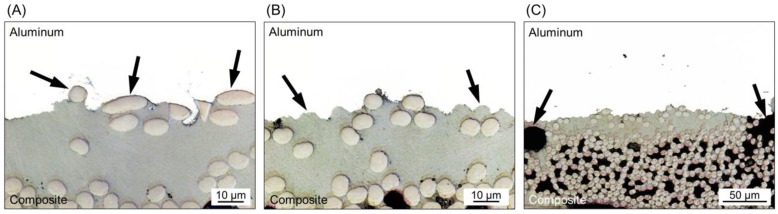
(**A**) Fiber anchoring (region a in Figure 5C), (**B**) PPS matrix entrapment (region b in Figure 5C) by the aluminum surface, and (**C**) volumetric defects in the composite part close to the joint’s interface (region b in Figure 5C).

A detailed analysis of the joint’s interface also revealed the sites of micro-mechanical interlocking between the crevices of aluminum and the consolidated composite matrix. As previously discussed, a thin layer of the PPS matrix close to the joint’s interface is melted during the joining process. The molten PPS is displaced from the center to the edges of the joint due to the axial force applied by the tool and the plastic deformation of the metal. Such displacement of the PPS matrix exposes some carbon fibers at the center of the joints to be in intimate contact with the aluminum surface. Figure 6A shows the presence of these fibers anchored by the irregularities of the sandblasted aluminum surface. The entrapment of the PPS matrix into the crevices of the aluminum surface was also identified, as shown in Figure 6B. It is possible to note that an effective micro-mechanical interlocking was achieved because the PPS matrix took the shape of the irregularities of the aluminum surface, while some fibers were entrapped into the crevices.

Figure 6C shows the presence of volumetric defects in the composite part close to the joint’s interface. It is believed that such defects are micro-voids generated by air entrapment due to the outflow of the molten matrix during the joining process. Some of these voids may also be correlated with the differential shrinkage between the metal and the composite matrix during joint consolidation [19]. The presence of microvoids was also addressed for AA2024-T3/CF-PPS friction spot joints by Goushegir et al. [16]. As mentioned previously, the maximum process temperature achieved for the joints in this study was 331 ± 4 °C. This temperature is far below the onset degradation temperature of PPS (for cross-linking 500 °C). Therefore, it is not expected that such voids are the result of the thermo-mechanical degradation of the composite part. 

**Figure 7:**
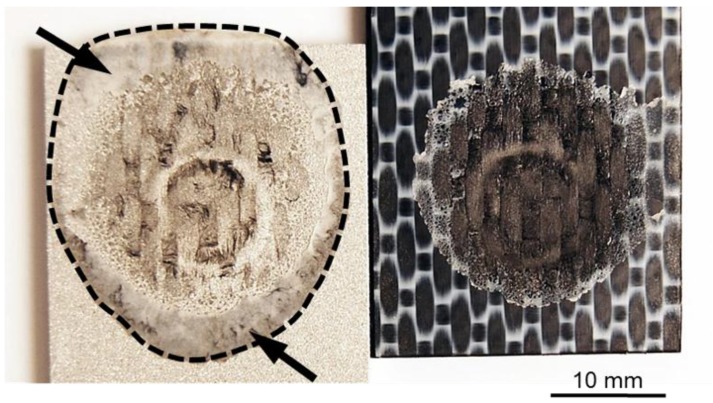
A representative example of the fracture surface of a friction spot joint. The arrows indicate the layer of the reconsolidated molten PPS.

As previously discussed, the generated frictional heat is conducted through the aluminum surface and melts a thin layer of the polymer matrix close to the joint’s interface. Owing to the axial force applied by the tool, the molten polymer is displaced from the center of the joint toward the edges of the overlap area (Figure 7). The layer of molten polymer reconsolidates during the cooling phase of the process, thereby establishing adhesion forces between the aluminum and composite. The bonding area in the friction spot joints can be determined by the perimeter of the reconsolidated molten polymer layer, as indicated by the dashed line in Figure 7.

Therefore, three main bonding mechanisms can be identified for AA7075-T6/CF-PPS friction spot joints: macro- and micro-mechanical interlocking and adhesion forces. Similar mechanisms were addressed for AA2024-T3/CF-PPS [16] and AA6181-T6/CF-PPS [15] friction spot joints.

### 4.3. Quasi-static Mechanical Performance

**Figure 8:**
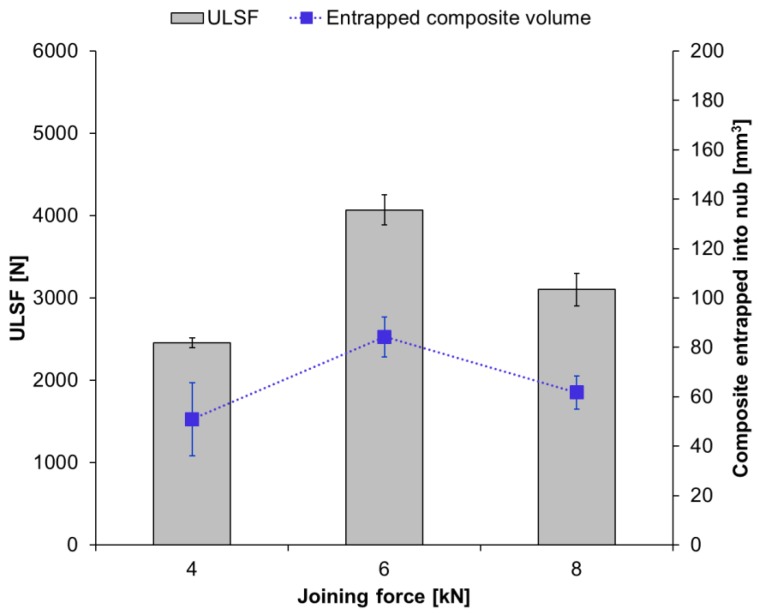
Average ultimate lap shear force of the joints along with the volume of the composite entrapped into the nub by using different joining forces (constant joining parameters RS: 1900 rpm, PD: 0.8 mm, JT: 4 s).

It can be seen that the ULSF of the joints does not show a linear correlation with the applied joining force (Figure 8). The strongest joint was obtained with intermediate joining force (JF: 6 kN; ULSF: 4068 ± 184 N). The joint produced with a low joining force resulted in a ULSF that is about 40% lower (JF: 4 kN; ULSF: 2456 ± 60 N), while the joint produced with a high joining force showed an approximately 24% lower ULSF (JF: 8 kN; ULSF: 3102 ± 199 N) than the ULSF obtained for with intermediate joining force.

**Figure 9:**
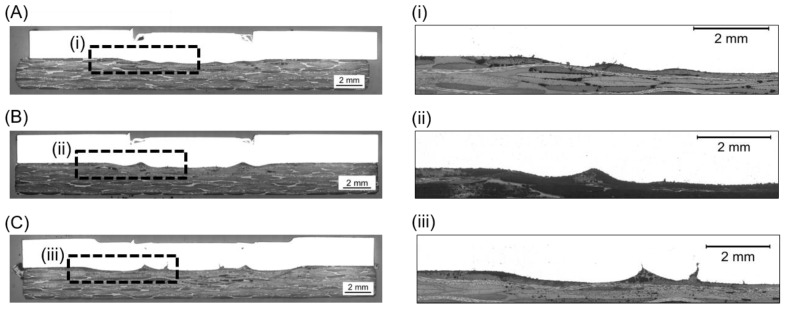
Cross-sections of friction spot joints produced with (**A**) 4 kN, (**B**) 6 kN, and (**C**) 8 kN. Details of the metallic nubs are given in (**i**), (**ii**) and (**iii**) for the joints produced with 4, 6 and 8 kN respectively (constant joining parameters RS: 1900 rpm, PD: 0.8 mm, JT: 4 s).

**Figure 10:**
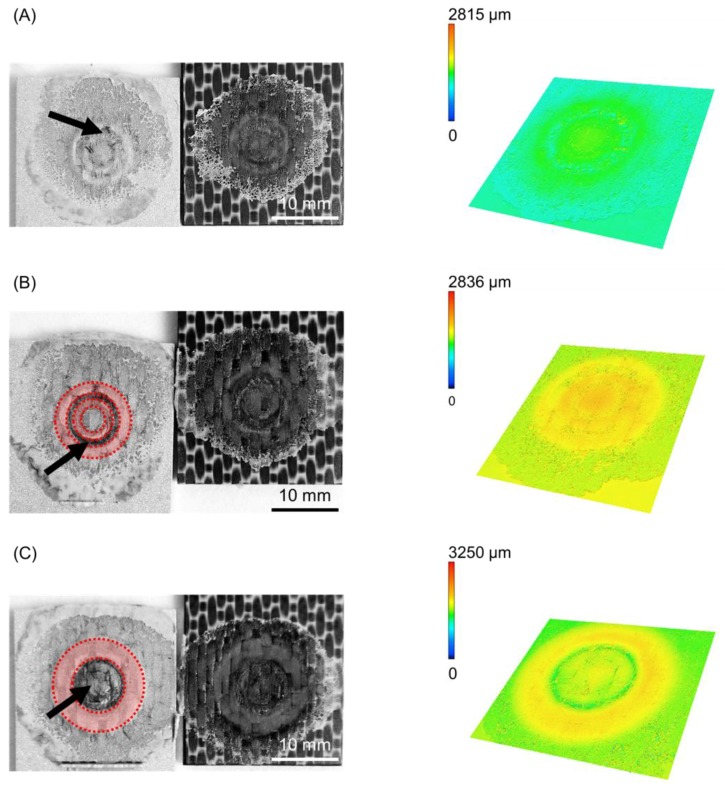
Fracture surface and 3D image of the deformation on the aluminum part (nub region) for the joints produced with (**A**) 4 kN, (**B**) 6 kN and (**C**) 8 kN of joining force (constant joining parameters RS: 1900 rpm, PD: 0.8 mm, JT: 4 s).

The cross-section of the joints produced by using various joining forces are presented in Figure 9. Furthermore, the fracture surface and respective 3D images of the metallic nub (obtained from the fracture surface of the joints on the aluminum side) are also presented in Figure 10. Different geometries of the metallic nub can be identified in the images. In the joint produced with low joining force (4 kN), the deformation of the aluminum into the composite was very shallow (the metallic nub, Figure 9A). Thus, the macro-mechanical interlocking between the aluminum and the composite is less effective. In this case, the volume of the composite entrapped into the nub was 51 ± 15 mm^3^ (Figure 10A) and the joints reached the lowest ultimate lap shear force (2456 ± 60 N) for the joining conditions studied in this work. The joint produced with the intermediate joining force (6 kN) presented a more pronounced deformation of the aluminum into the composite (Figure 9B). In this case, the deformation of the aluminum into the composite retained the shape of two rings (ellipses in Figure 10B). This geometry provides two sites of macro-mechanical interlocking between the aluminum and composite, thereby maximizing the volume of the composite entrapped into the nub (84 ± 8 mm^3^). Therefore, the highest mechanical performance of the joints (4068 ± 184 N) was achieved in this study. The aluminum deformation in the joint produced with high joining force (8 kN) resulted in the shape with only one wide ring (Figure 9C). Such geometry provides only one site for the macro-mechanical interlocking between the aluminum and the composite (Figure 10C). Moreover, the volume of the composite entrapped into the nub was 62 ± 7 mm^3^. Therefore, a decrease in the ULSF was observed for the joint produced with 8 kN (3102 ± 199 N) compared to those produced with 6 kN.

It is worth noting, that for all the investigated joints, a layer of the reconsolidated molten PPS was formed and remained attached to the aluminum surface (Figure 10), providing adhesion forces. Additionally, signs of fiber and matrix entrapment on the aluminum surface were also observed in all cases (black arrows in Figure 10). These results indicate the importance of the nub geometry and its influence on the macro-mechanical interlocking between the joining parts and hence the mechanical performance of the friction spot joints. Further investigation using the finite element method (FEM) may help to better understand the influence of the geometry of the metallic nub on the mechanical strength of the friction spot joints.

**Figure 11:**
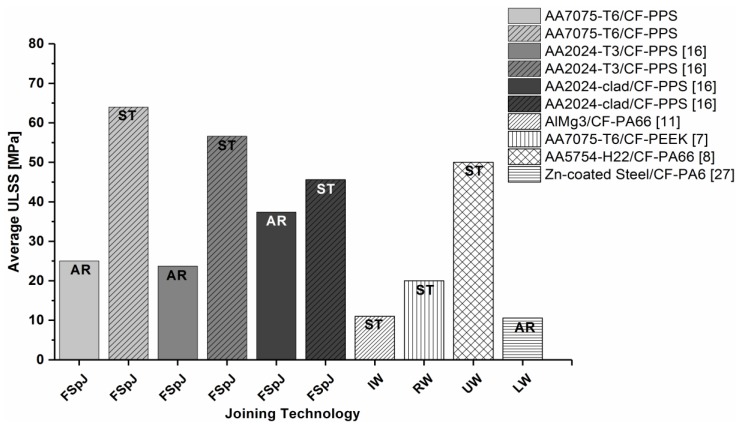
Qualitative comparison of the ultimate lap shear strength (ULSS) of the state-of-the-art and concurrent joining technologies for hybrid structures (AR: as received, ST: surface treatment).

A qualitative comparison between the state-of-the-art welding-based joining technologies for metal–polymer hybrid structures is given in Figure 11. Induction welding (IW) [11], resistance welding (RW) [7], ultrasonic welding (UW) [8], and laser welding (LW) [27] were included in the comparison. Joints with similar materials (metals and carbon-fiber-reinforced polymers), configuration (overlap), surface pre-treatments, thicknesses, and failure mechanisms to friction spot joints were chosen. Figure 11 shows that the friction spot joints presented comparable or superior quasi-static strength to those provided by the concurrent technologies. Another advantage of FSpJ is the process time. The friction spot joints are produced in a single-step joining cycle, which is performed in a few seconds (4 s in this study). However, for example, the induction welding process lasts about 1 min [11], while the resistance welding process can take from 30 s up to 5 min [7]. The ultrasonic welding also has a joining cycle similar to FSpJ (3.5 s) [8].

### 4.4. Fracture Mechanisms

**Figure 12:**
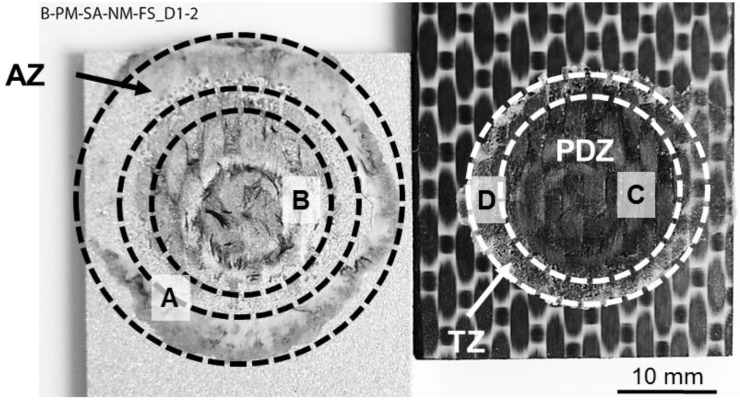
Typical fracture surface of AA7075-T6/CF-PPS friction spot joints along with the defined bonding zones. The regions analyzed by SEM are indicated as A–D.

The fracture mechanisms of the joints were investigated through a detailed SEM analysis of their fracture surface after lap shear testing. Three bonding zones were identified: Plastically Deformed Zone (PDZ), Transition Zone (TZ), and Adhesion Zone (AZ), as previously described by Goushegir et al. [16]. Figure 12 shows a typical fracture surface of AA7075-T6/CF-PPS friction spot joints along with the defined bonding zones.

**Figure 13:**
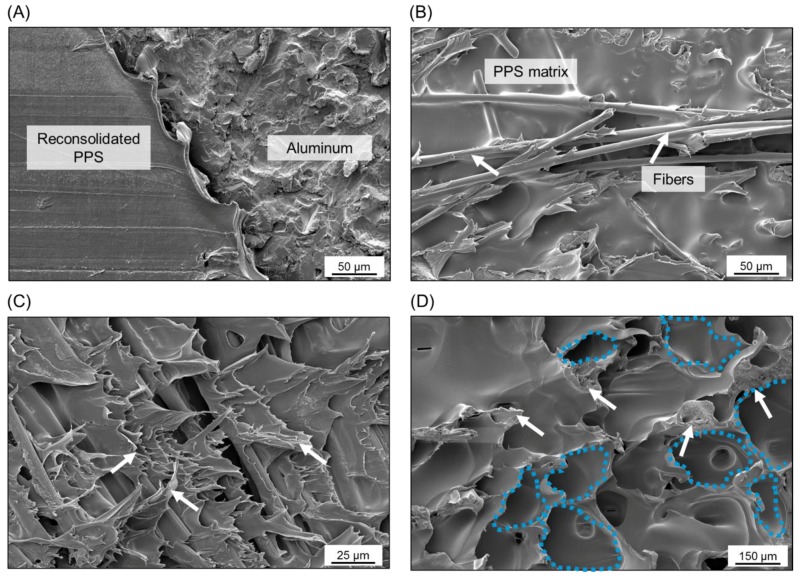
Detailed SEM images of the bonding zones in a representative AA7075-T6/CF-PPS friction spot joint. (**A**) The transition between AZ and TZ. (**B**) The PDZ on the aluminum surface. (**C**) The PDZ and (**D**) the TZ on the composite surface.

AZ is the external region of the bonding area originated from the layer of reconsolidated molten polymer expelled from the center of the joint during the joining process. In this zone, the main bonding mechanism is the adhesion forces provided by the reconsolidation of the molten polymer matrix in contact with the aluminum surface. Figure 13A shows a featureless fracture surface of the AZ (reconsolidated molten PPS). It indicates that the failure occurred in this zone due to the detachment of the reconsolidated molten PPS from the composite surface, thus characterizing an adhesive failure mode.

PDZ is the central region of the bonding area where the metallic nub is formed. In this zone, the highest process temperatures are achieved due to the proximity to the tool. The plastic deformation of the metal into the composite displaces a volume of the softened/melted composite’s matrix, thus exposing the carbon fibers on the surface of the composite. Therefore, micro-mechanical interlocking (in the form of PPS and carbon-fiber entrapment into the aluminum surface) was identified in this region as previously discussed (Figure 6A,B). Figure 13B shows that the carbon fibers and the PPS matrix remained attached to the aluminum surface after the failure of the joint by mechanical testing. This feature indicates an effective micro-mechanical interlocking in this zone between the aluminum and composite. Additionally, this residual composite material on the aluminum surface indicates that the crack propagated in this zone through the first plies of the composite part instead of at the interface. Figure 13C shows that the fibrils of the PPS originated from the large plastic deformation of the composite’s matrix during failure. Therefore, the failure occurred in the PDZ through a cohesive failure mode with a predominantly ductile micro-mechanism of failure. Such a ductile micro-mechanism was also reported for metal–composite hybrid joints welded by Resistance Welding [10] and Ultrasonic Welding [8].

TZ is the transition region between AZ and PDZ. This zone is characterized by the presence of air bubbles formed during the displacement of the molten matrix from the center to the edge of the overlap area during the joining process (see discussion in Section 4.2). Figure 13D shows the air bubbles in this zone on the composite surface. The white arrows indicate plastic deformation sites and tearing of the PPS around the bubbles. It suggests that the cohesive failure mode in the TZ is predominant and that a mixture of brittle and ductile micro-mechanisms of failure occurred. A similar fracture micro-mechanism was also observed for AA2024-T3/CF-PPS friction spot joints in [28].

## 5. Conclusions

Friction spot joints of aluminum alloy 7075-T6 and carbon-fiber-reinforced polyphenylene sulfide (CF-PPS) were produced and evaluated for the first time in the literature. The main conclusions drawn from this work are:

- Three main bonding mechanisms were identified at the metal–composite interface: macro- and micro-mechanical interlocking and adhesion forces. The macro-mechanical interlocking was provided by the plastic deformation of the aluminum (metallic nub) into the composite part. The micro-mechanical interlocking at the metal–composite interface was provided by the entrapment of the PPS matrix and carbon fibers into the aluminum surface. Additionally, the reconsolidated molten PPS matrix led to the adhesion forces between the joining parts.
- Ultimate lap shear force of up to 4068 ± 184 N was achieved in this study. The joining force showed a significant influence on the nub geometry and hence on the ULSF of the joints. Intermediate joining force (6 kN in this study) originated a metallic deformation in the shape of two rings inserted into the composite part. This geometry effectively interlocked the aluminum and the composite part, thereby maximizing the volume of the composite entrapped into the nub (84 ± 8 mm^3^) and consequently the ULSF of the joint.
- A qualitative comparison with other state-of-the-art joining technologies for hybrid structures demonstrated that the friction spot joints exhibit superior/similar strength than/to the concurrent joining technologies for hybrid structures.
- The fracture surface of the joints showed that the bonding area could be divided into different zones. Three bonding zones were identified as the following: Plastically Deformed Zone (PDZ), Transition Zone (TZ), and Adhesion Zone (AZ), as previously reported in the literature for other combinations of materials joined with FSpJ.
- A mixture of adhesive–cohesive failure mode was identified, while cohesive failure was dominant. A combination of brittle and ductile micro-mechanisms of failure was observed by SEM analysis.

In face of the findings of this work, further investigations regarding the influence of the nub geometry on the mechanical performance of the joints, as well as the assessment of the fatigue performance of such structures for the transportation industry are required. This will be the focus of the coming publications of the group.

## Figures and Tables

**Table 1:** Nominal chemical composition of AA7075-T6 [20].

Element	Zn	Mg	Cu	Fe	Si	Mn	Cr	Ti	Al
Wt.%	6.1–5.1	2.1–2.9	1.2–2.0	0.50	0.40	0.30	0.18–0.28	0.2	Bal.

**Table 2:** Selected physical and mechanical properties of AA7075-T6 [21].

Tensile Strength (TL * direction) (MPa)	Yield Strength (TL * direction) (MPa)	Elongation (%)	Incipient Melting Temperature (°C)	Thermal Conductivity (W m^−1^ K^−1^)	Coefficient of Thermal Expansion, 20–300°C (µm m^−1^ °C^−1^)
538	469	8	477	130	25.2

* TL: transverse to lamination.

**Table 3:** Selected physical and mechanical properties of CF-PPS [22].

Tensile Strength (warp/weft) (MPa)	In-Plane Shear Strength (MPa)	Glass Transition Temperature—T_g_ (°C)	Melting Temperature—T_m_ (°C)	Thermal Conductivity (W m^−1^ K^−1^)	Coefficient of Thermal Expansion, 23–300 °C (µm m^−1^ °C^−1^)
790/750	119	120	280	0.19	52.2

**Table 4:** Joining parameters used in the current study.

Condition	Rotational Speed (rpm)	Plunge Depth (mm)	Joining Time (s)	Joining Force (kN)
C1	1900	0.8	4	4
C2	1900	0.8	4	6
C3	1900	0.8	4	8
